# Return of Spontaneous Circulation Depends on Cardiac Rhythm During Neonatal Cardiac Arrest in Asphyxiated Newborn Animals

**DOI:** 10.3389/fped.2021.641132

**Published:** 2021-02-12

**Authors:** Michael Wagner, Po-Yin Cheung, Maryna Yaskina, Tze-Fun Lee, Vanessa A. Vieth, Megan O'Reilly, Georg M. Schmölzer

**Affiliations:** ^1^Division of Neonatology, Pediatric Intensive Care and Neuropediatrics, Department of Pediatrics, Comprehensive Center for Pediatrics, Medical University of Vienna, Vienna, Austria; ^2^Neonatal Research Unit, Centre for the Studies of Asphyxia and Resuscitation, Royal Alexandra Hospital, Edmonton, AB, Canada; ^3^Department of Pediatrics, University of Alberta, Edmonton, AB, Canada; ^4^Division of Neonatology, Department of Pediatrics, Medical University Graz, Graz, Austria

**Keywords:** cardiac rhythm, chest compressions, neonatology, resuscitation, sustained inflation

## Abstract

**Objective:** Pulseless electrical activity (PEA) occurs in asphyxiated newborn piglets and infants. We aimed to examine whether different cardiac rhythms (asystole, bradycardia, PEA) affects the resuscitation outcomes during continuous chest compressions (CC) during sustained inflations (CC+SI).

**Design:** This study is a secondary analysis of four previous randomized controlled animal trials that compared CC+SI with different CC rate (90 or 120/min), SI duration (20 or 60 s), peak inflation pressure (10, 20, or 30 cmH_2_O), and oxygen concentration (18, 21, or 100%).

**Setting and Subjects:** Sixty-six newborn mixed breed piglets (1–3 days of age, weight 1.7–2.4 kg) were obtained on the day of experimentation from the University Swine Research Technology Center.

**Interventions:** In all four studies, piglets were randomized into intervention or sham. Piglets randomized to “intervention” underwent both hypoxia and asphyxia, whereas, piglets randomized to “sham” received the same surgical protocol, stabilization, and equivalent experimental periods without hypoxia and asphyxia.

**Measurements:** To compare differences in asphyxiation time, time to return of spontaneous circulation (ROSC), hemodynamics, and survival rate in newborn piglets with asystole, bradycardia or PEA.

**Main Results:** Piglets with PEA (*n* = 29) and asystole (*n* = 13) had a significantly longer asphyxiation time and time to ROSC vs. bradycardia (*n* = 24). Survival rates were similar between all groups. Compared to their baseline, mean arterial pressure and carotid blood flow were significantly lower 4 h after resuscitation in all groups, while being significantly higher in the bradycardia group.

**Conclusion:** This study indicates that cardiac rhythm before resuscitation influences the time to ROSC and hemodynamic recovery after ROSC.

## Introduction

At birth, the clinical team assesses the infant's heart rate (HR) to guide intervention during neonatal resuscitation (1). If HR is <60/min, chest compressions (CC) must be started (1) Bradycardia and asystole were believed to be the most common cardiac arrest rhythms in newborn infants (2). However, there is a lack of data what HR cut-off to start CC should be used in neonatal patients with bradycardia secondary to asphyxia. Most recently, studies have reported that pulseless electrical activity (PEA) is commonly observed in asphyxiated newborn piglets and infants (3–6). PEA displays an organized cardia rhythm on the electrocardiogram (ECG) without cardiac output or essential blood flow, and is mostly caused by hypoxia and hypovolemia (4).

If a newborn requires chest compression, the neonatal resuscitation guideline recommends a 3:1 Compression:Ventilation (C:V) ratio (1, 7, 8). An alternative approach of neonatal chest compression, which combines CC during continuous sustained inflation (SI) (providing constant high airway pressure during CC=CC+SI) has been described by our group (9–13). The CC+SI approach significantly improved systemic and regional hemodynamics, tidal volume delivery, minute ventilation, and time to return of spontaneous circulation (ROSC) compared to 3:1 C:V in neonatal piglets (14).

During adult cardiac arrest, the presenting cardiac rhythm is most often (81%) non-shockable (i.e., asystole or PEA) (15). In pediatric patients, the presenting cardiac arrest rhythms are asystole (58%), bradycardia (6%), PEA (15%), ventricular fibrillation or tachycardia (8%), and unknown rhythms (13%) (16). In comparison, there is a lack of data about the presenting cardiac arrest rhythm in newborns in the delivery room. Kumar et al. (17) reported 50 infants with asystole and 160 infants with bradycardia over a 16-year period (17). Overall, the time to return of spontaneous circulation (ROSC) was 12.5 min compared to 7 min in infants with asystole vs. bradycardia. There is limited evidence about outcomes of PEA in newborns. Therefore, we aimed to examine the outcomes of neonatal resuscitation and recovery depending on the presenting cardiac rhythm (i.e., bradycardia vs. PEA vs. asystole) in asphyxiated newborn piglets.

## Materials and Methods

This is a secondary analysis of our four previous randomized controlled animal trials, which examined CC+SI. Only data from piglets resuscitated with CC+SI were examined.

To optimize the effectiveness of CC+SI in resuscitation, series of experiments were carried out to examine whether the outcome can be improved by alternating the CC rate (90 or 120/min) (Study 1)(11), SI duration (20 or 60 s) (Study 2) (18), inflation pressure (10, 20, or 30 cmH_2_O) (Study 3) (19) or oxygen concentration (18, 21, or 100%) (Study 4) (20). As no difference was observed among groups with various interventions and the experimental protocols as well as piglets breed were also similar within all these studies, all data were combined for secondary analyses.

**Figure 1:**
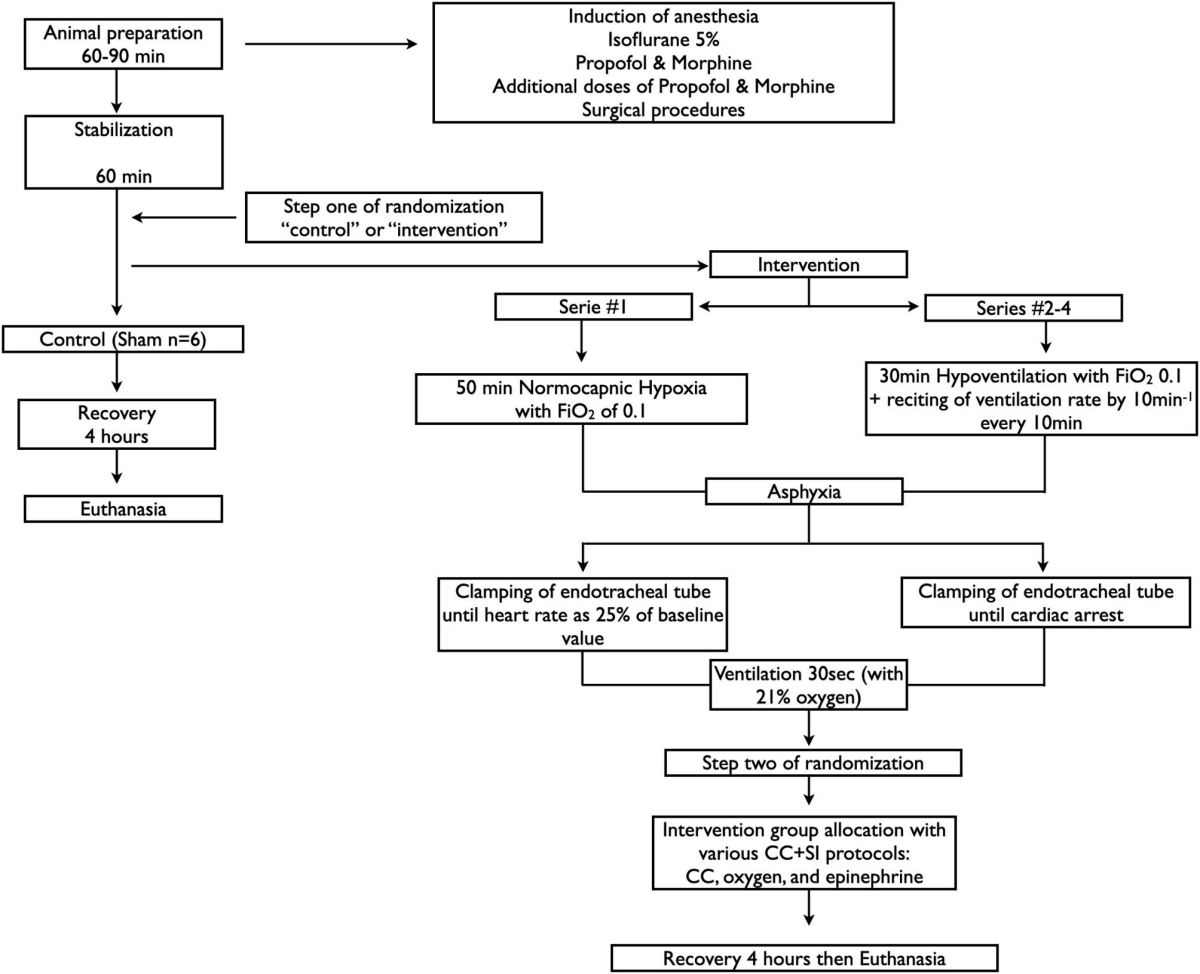
Study flow diagram.

Sixty-six newborn mixed breed piglets (1–3 days of age, weighing 1.7–2.4 kg) were obtained on the day of experimentation from the University Swine Research Technology Center. All experiments were conducted in accordance with the guidelines and approval of the Animal Care and Use Committee (Health Sciences), University of Alberta (AUP00001764, AUP00002151, and AUP00002651), presented according to the ARRIVE guidelines (21) and registered at preclinicaltrials.eu (PCTE0000138). A graphical display of the study protocol is presented in Figure 1.

### Animal Preparation

Following the induction of anesthesia using isoflurane, piglets were intubated via a tracheostomy, and pressure-controlled ventilation (Sechrist infant ventilator model IV-100; Anaheim, USA for series 1 and Acutronic Fabian HFO; Hirzel, Switzerland for series 2–4) was commenced at a respiratory rate of 16–20 breaths/min and pressure of 20/5 cmH_2_O. Oxygen saturation was kept within 90–100%, glucose level and hydration were maintained with an intravenous infusion of 5% dextrose at 10 mL/kg/hr. During the experiment, anesthesia was maintained with intravenous propofol (5–10 mg/kg/h) and morphine (0.1 mg/kg/h). Additional doses of propofol (1–2 mg/kg) and morphine (0.05–0.1 mg/kg) were given as needed. Only in series 1, pancuronium (0.1–0.2 mg/kg) was used for controlling the arterial CO_2_ level. The piglet's body temperature was maintained at 38.5–39.5°C using an overhead warmer and a heating pad (9, 14, 22).

### Hemodynamic Parameters

A 5-French Argyle^®^ (Klein-Baker Medical Inc., San Antonio, TX) double-lumen catheter was inserted via the right femoral vein for administration of fluids and medications. A 5-French Argyle^®^ single-lumen catheter was inserted above the right renal artery via the femoral artery for continuous arterial blood pressure monitoring in addition to arterial blood gas measurements. The right common carotid artery was exposed and encircled with a real-time ultrasonic flow probe (2 mm; Transonic Systems Inc., Ithica, NY) to measure carotid arterial blood flow (CABF). Piglets were placed in supine position and allowed to recover from surgical instrumentation until baseline hemodynamic measures were stable (minimum of 1 h). Ventilator rate was adjusted to keep the partial arterial CO_2_ pressure between 35 and 45 mmHg, as determined by periodic arterial blood gas analysis. Baseline blood gas values were obtained after stabilization and just before hypoxia. Mean systemic arterial pressure, HR, and percutaneous oxygen saturation were continuously measured and recorded throughout the experiment with a Hewlett Packard 78833B monitor (Hewlett Packard Co., Palo Alto, CA).

### Cerebral Perfusion

Cerebral oxygenation was measured using the Invos Cerebral/Somatic Oximeter Monitor (Invos 5100, Somanetics Corp., Troy, MI). The sensors were placed on the right forehead of the piglet and secured with wrap and tape. Light shielding was achieved with a slim cap. The Invos Cerebral/Somatic Oximeter Monitor calculates cerebral oxygenation, which is expressed as the percentage of oxygenated hemoglobin (oxygenated hemoglobin/total hemoglobin). Values of regional oxygen saturation are stored every second with a sample rate of 0.13 Hz (23).

### Experimental Protocol

In all four studies, piglets were randomized into intervention or sham. To reduce selection bias, a two-step randomization process was used. Following surgical instrumentation and stabilization, a subsequently numbered, sealed opaque envelope containing the assignment “sham” or “intervention” was opened (step one) (Figure 1). Piglets randomized to “intervention” underwent both hypoxia and asphyxia, whereas, piglets randomized to “sham” did not. Sham-operated groups received the same surgical protocol, stabilization, and equivalent experimental periods without hypoxia and asphyxia. The piglets that were randomized to “intervention” were exposed to either 30 min (asystole group, Studies 2–4, (18)) or 50 min (bradycardia group, Study 1 (11)) of normocapnic hypoxia, which was followed by asphyxia. Asphyxia was achieved by disconnecting the ventilator and clamping the endotracheal tube to a pre-set end-point. Study 1: Asphyxia was induced until bradycardia (defined as a decrease in HR to 25% of baseline) (11). Studies 2–4: Asphyxia was induced until asystole (defined as no audible HR during continuous auscultation and zero carotid artery blood flow) (18–20). PEA was defined as zero HR but with electrical activity on the ECG (3, 4). After the end-point was reached, a second subsequently numbered, sealed opaque envelope containing the intervention assignment was opened (step two) (Figure 1). Fifteen seconds after bradycardia or asystole, positive pressure ventilation was performed for 30 s with a Neopuff T-Piece (Fisher & Paykel, Auckland, New Zealand). The default settings of the experiment were a peak inflating pressure of 30 cmH_2_O (except 16 piglets in study 3, which were randomized to peak inflating pressure of either 10 or 20 cm H_2_O), a positive end expiratory pressure (PEEP) of 5 cmH_2_O, and a gas flow of 8 L/min. Using the two-thumb encircling technique (7), CC was performed at a rate of 90/min (except eight piglets with rate of 120/min in study 1) using a metronome by a single operator in all the piglets. CC was started after 30 s of positive pressure ventilation and 100% oxygen was commenced 30 s after start of CC. Epinephrine (0.02 mg/kg per dose) was administered intravenously 2 min after the start of positive pressure ventilation, and administered every 3 min as needed if no ROSC was observed. Epinephrine was administered to a maximum of 4 doses as the maximum resuscitation time was set at 12 min. ROSC was defined as an unassisted HR > 100 bpm for at least 15 s. After ROSC, piglets recovered for 4 h before euthanasia with an intravenous overdose of phenobarbital (100 mg/kg).

### Data Collection and Analysis

Demographics of study piglets were recorded. Transonic flow probes, HR, and pressure transducer outputs were digitized and recorded with LabChart^®^ programming software (ADInstruments, Houston, TX). Data are presented as mean (standard deviation) for normally distributed continuous variables and median (interquartile range) when the distribution was skewed. The data was tested for normality and compared using one-way ANOVA for comparisons of continuous variables, and χ^2^ for categorical variables. *Post-hoc* analysis was performed using Tukey test. Time to ROSC was analyzed with Cox proportional hazards regression by using SAS Proc SURVEYPHREG with stratification by studies, to account for data being combined from different trials. The event was considered as ROSC. Piglets who did not achieve ROSC were considered censored at a maximum of 12 min (720 s) of cardiopulmonary Resuscitation time. The proportionality assumption was violated for bradycardia and asystole groups by assessment of Kaplan–Meier and Log–Log curves. Therefore, three different Cox proportional hazards regression models were created for pairwise comparison of PEA, bradycardia and asystole groups. Both models for PEA and asystole groups, and PEA and bradycardia groups satisfied proportionality assumption; however, since this assumption was violated for bradycardia and asystole groups, the interaction term (cardiac group)*(time to ROSC) was added to the Cox regression model for those 2 groups. Both terms from that model, cardiac group and interaction (cardiac group)*(time to ROSC), were used to estimate the hazard ratio, which was not constant over time. Statistical analyses were performed with SigmaPlot (Systat Sofyware Inc., San Jose) and SAS Ver. 9.4 (SAS Institute Inc., Cary, NC, USA).

## Results

Age and sex were similar among the three experimental groups (bradycardia, asystole, PEA) (Table 1). However, baseline hemodynamic and blood gas values were significantly better in the bradycardia group compared to the PEA and asystole groups (Table 1). These differences were most likely related to the different surgical procedures, experimental set-up, and medication (10). Therefore, we compared changes from baseline for hemodynamic parameters within the same group.

**Table 1:** Baseline characteristics.

	**Bradycardia (*n* = 24)**	**PEA (*n* = 29)**	**Asystole (*n* = 13)**	***p*-value**
Age (days)	2.0 (2.0 to 3.0)	2.0 (1.0 to 3.0)	2.0 (1.0 to 2.0)	0.284
Sex (male/female)	15/9	18/11	7/6	0.894
paCO_2_ (mmHg)	40 (36 to 42)	34 (30 to 35)^*^	33 (29 to 35)^*^	<0.001
pH	7.37 (7.34 to 7.42)	7.49 (7.46 to 7.54)^*^	7.55 (7.47 to 7.58)^*^	<0.001
BE (mmol/L)	-2 (-6 to 0)	2 (1 to 4)^*^	4 (2 to 5)^*^	<0.001
${\text{HCO}}_{3}^{-}$ (mmol/L)	24 (21 to 25)	25 (25 to 27)^*^	27 (25 to 29)^*^	0.002
Lactate (mmol/L)	3.6 (2.7 to 4.1)	3.6 (2.9 to 4.9)	3.2 (3.0 to 3.5)	0.280
Heart rate (bpm)	228 (215 to 248)	193 (173 to 211)^*^	186 (160 to 202)^*^	<0.001
Mean arterial pressure (mmHg)	77 (69 to 87)	60 (56 to 66)^*^	57 (53 to 63)^*^	<0.001
Carotid flow (mL/min)	77 (65 to 90)	47 (40 to 56)^*^	45 (33 to 51)^*^	<0.001
Cerebral oxygenation index (%)	41 (39 to 47)	53 (48 to 57)^*^	49 (42 to 58)^*^	<0.001

Data are presented as median (IQR);

* *significantly different from bradycardia group. PEA, pulsless electrical activity*.

### Resuscitation

The asphyxia time was significantly longer in the PEA and asystole groups compared to the bradycardia group (Table 2). This resulted in significantly lower pH, higher paCO_2_, and lactate in both groups compared to the bradycardia group (Table 2).

**Table 2:** Characteristics of asphyxia, resuscitation, and survival of asphyxiated piglets.

	**Bradycardia (*n* = 24)**	**PEA (*n* = 29)**	**Asystole (*n* = 13)**	***p*-value**
PaCO_2_	67 (58 to 85)	98 (87 to 113)^*^	116 (104 to 122)^*^	<0.001
pH	6.91 (6.83 to 6.98)	6.59 (6.50 to 6.67)^*^	6.50 (6.50 to 6.54)^*^	<0.001
BE (mmol/L)	-20 (-22 to -16)	-27 (-30 to -26)^*^	-30 (-30 to -28)^*^	<0.001
Lactate (mmol/L)	13.0 (11.7 to 14.1)	15.8 (14.3 to 17.1)^*^	15.7 (14.7 to 16.2)^*^	<0.001
Asphyxiation time (s)	112 (69 to 180)	475 (221 to 589)^*^	332 (260 to 460)^*^	<0.001
ROSC time (s)	35 (28 to 68)	120 (80 to 175)^*^	69 (60 to 111)	<0.001
Number of piglets with ROSC	20 (83%)	16 (55%)^*^^#^	13 (100%)	<0.01
Total number of Epi dose	0 (0 to 3)	1 (0 to 4)^#^	0 (0 to 0.5)	0.004
Number of piglets receiving Epi	10 (42%)	20 (69%)^#^	3 (23%)	<0.01
Overall survival after resuscitation	20 (100%)	16 (100%)	12 (92%)	0.26

Data are presented as median (IQR); Epi, epinephrine;

* Significantly different from bradycardia group;

# *Significantly different from asystole group. PEA, pulsless electrical activity*.

Time to ROSC was significantly shorter in the bradycardia group compared to the asystole and PEA group (Table 2). The rate of ROSC was significantly lower in the PEA group compared to the bradycardia and asystole groups (Table 2). The total number of epinephrine doses and the number of piglets receiving epinephrine were significantly higher in the PEA group compared to the asystole group (Table 2). There was no significant difference in post-ROSC survival between all three groups (Table 2). In Cox regression models the hazard ratios between subjects from different cardiac groups are presented in Table 3. For bradycardia and asystole groups hazard ratio depended on time. As time increased, the hazard ratio increased as well.

**Table 3:** Cox Regression estimates of hazard ratios.

**Model**		**Estimate**	**SE**	***p*-value**	**Univariate hazard ratio (95% CI)**
Asystole vs. PEA		1.51	0.34	<0.0001	4.53 (2.29, 8.97)
Bradycardia vs. PEA		1.12	0.33	0.002	3.05 (1.58, 5.91)
Bradycardia vs. Asystole	Bradycardia vs. Asystole	1.96	0.71	0.01	7.07 (1.65, 30.34)
	Cardiac group^*^time to ROSC (interaction)	0.03	0.01	0.02	1.03 (1.003, 1.05)

### Changes in Hemodynamic Parameters

**Figure 2:**
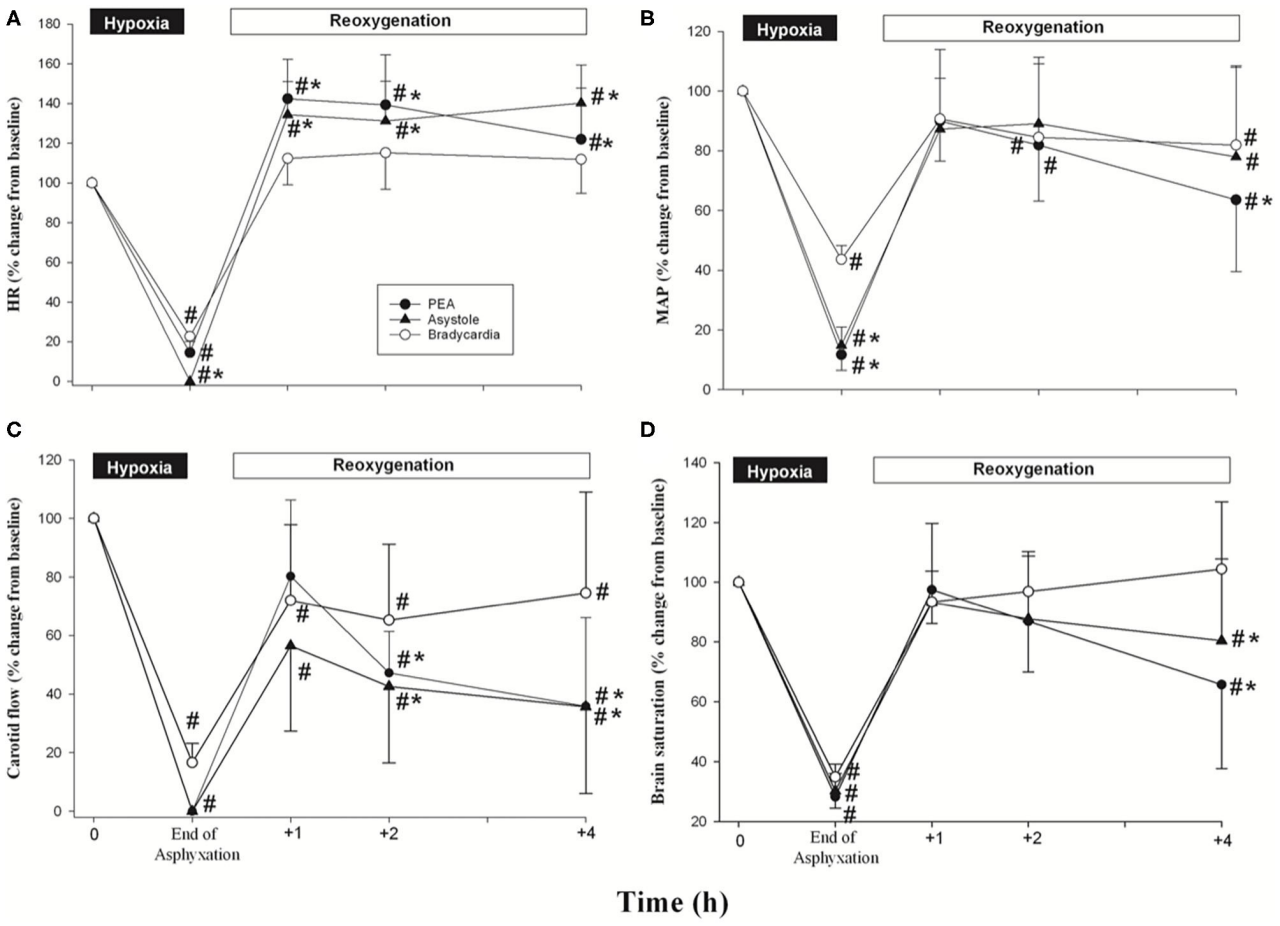
Percentage changes from normoxic baseline in **(A)** heart rate, **(B)** mean arterial pressure (MAP), **(C)** carotid blood flow, and **(D)** brain saturation in bradycardia (◦), PEA (•), and asystole (▴) groups during hypoxia/asphyxia and after resuscitation. Each point represents mean ± SD. *p < 0.05, significantly different from bradycardia group; ^#^*p* < 0.05, significantly different from its own baseline value.

Hemodynamic changes throughout the experiment are summarized in Figure 2. Heart rate significantly decreased at the end of asphyxia compared to baseline in all three groups, and returned to baseline after ROSC, with significantly higher HR in the PEA and asystole groups than that of bradycardia group (Figure 2).

The mean arterial blood pressure decreased significantly from baseline values after asphyxia (Figure 2), returned to baseline after ROSC, and then gradually decreased over the 4 h observation period. Compared to baseline, all groups had a significantly lower mean arterial pressure at the end of the 4 h observation period (Figure 2). The mean arterial pressure in the PEA group was significantly lower compared to the bradycardia group (Figure 2).

At the end of asphyxia, the CABF significantly decreased in all three groups compared to baseline (Figure 2). At the end of the 4 h observation period, the CABF remained significantly lower compared to baseline. Of note, the CABF was significantly lower in the PEA and asystole groups compared to the bradycardia group at the end of the 4 h observation period [36(30)%, 36(30)%, and 75(35)% of baseline value for PEA, asystole and bradycardia group, respectively] (Figure 2). Consequently, the cerebral oxygenation in the PEA and asystole groups were also significantly lower compared to the bradycardia group at the end of the 4 h observation period (Figure 2).

### Changes in Blood Gas

The pH in all groups significantly decreased from baseline values after asphyxia (Table 4). It improved after ROSC and gradually increased throughout the 4 h observation period. At the end of the 4 h observation period, the pH in the bradycardia group was similar compared to baseline, whereas the pH in the PEA and asystole groups remained significantly lower than their baseline (Table 4). There were similar patterns of changes in base excess, bicarbonate, and lactate throughout the experiment (Table 4).

**Table 4:** Blood gas at baseline, after asphyxiation, and 4 h of reoxygenation.

	**Bradycardia**	**With PEA**	**Asystole**	**p-value**
**pH**				
Baseline	7.37 (0.07)	7.49 (0.06)^*^	7.53 (0.07)^*^	<0.001
After asphyxiation	6.90 (0.11)^#^	6.60 (0.09)^*^^#^	6.53 (0.05)^*^^#^	<0.001
4 h after reoxygenation	7.33 (0.07)	7.29 (0.10)^#^	7.36 (0.13)^#^	0.082
**PaCO_2_ (mmHg)**				
Baseline	42 (6)	34 (4)^*^	32 (2)^*^	<0.001
After asphyxiation	71 (19)^#^	100 (18)^*^^#^	114 (11)^*^^#^	<0.001
4 h after reoxygenation	41 (6)	37 (6)^*^	36 (6)^*^	<0.001
**Base excess (mmol/L)**				
Baseline	-2 (4)	2 (3)^*^	4 (4)^*^	<0.001
After asphyxiation	-19 (5)^#^	-28 (2)^*^^#^	-29 (2)^*^^#^	<0.001
4 h after reoxygenation	-4 (2)	-8 (5)^*^^#^	-5 (6)^#^	0.005
**HCO_3_ (mmol/L)**				
Baseline	24 (3)	26 (2)^*^	27 (3)^*^	0.002
After asphyxiation	12 (2)^#^	9 (3)^*^^#^	9 (4)^*^^#^	<0.001
4 h after reoxygenation	21 (3)	18 (4)^*^^#^	20 (4)^#^	0.014
**Lactate (mmol/L)**				
Baseline	3.7 (1.0)	4.1 (0.9)	3.6 (0.7)	0.353
After asphyxiation	12.9 (2.1)^#^	15.6 (2.2)^*^^#^	15.4 (2.7)^*^^#^	<0.001
4 h after reoxygenation	4.1 (2.4)	5.7 (3.3)^#^	4.5 (2.6)	0.121

Data are presented as mean (SD);

* *Significantly different from bradycardia group*,

# *Significantly different from baseline values. PEA, pulsless electrical activity*.

## Discussion

There is limited evidence about management of different cardiac arrest rhythms during neonatal resuscitation and their outcome (4–6, 24). While healthcare providers routinely recognize bradycardia and asystole in the delivery room, the detection of PEA rhythms has only been described recently (4). This is the first study that investigated the influence of different cardiac arrest rhythms on resuscitation outcomes and recovery in asphyxiated piglets resuscitated by different CC+SI approaches. Compared to both the bradycardia and asystole groups, the PEA group required a longer resuscitation time and had less animals achieving ROSC and overall survival. Furthermore, epinephrine was used more often in the PEA group compared to the two other experimental groups. Our results also demonstrated that the hemodynamic recovery of the bradycardia group was better than the PEA and asystole groups.

Recent animal studies (9, 11, 25, 26) and clinical observations (12) have indicated that CC+SI significantly improves ROSC, tidal volume delivery and minute ventilation, as well as global and regional hemodynamics compared to conventional 3:1 C:V. Currently, a large cluster randomized trial comparing CC+SI with 3:1 C:V in newborn infants is ongoing (SURV1VE) (13). Therefore, our study focused on the effect of CC+SI on resuscitation outcomes and hemodynamic recovery in asphyxiated piglets with different heart rhythms (bradycardia, PEA, or asystole). As indicated by lower pH, higher base excess and lactate levels, the hypoxia/asphyxia stress in both PEA and asystole groups were more severe than in the bradycardia group. Consequently, a prolonged time to ROSC, particularly in the PEA group, was observed compared to the bradycardia group. In a neonatal lamb asphyxia model lactate levels were also higher in animals with no ROSC (27). Further, the pH of these two groups remained lower throughout the experiment whereas the pH in the bradycardia group returned to baseline by the end of the 4 h observation period. These observations were similar to that reported following resuscitation of newborn infants with bradycardia or asystole (17). In that study, asystole was associated with a lower cord blood pH and a lower pH 1 h after birth, and infants required significantly more intubations and CCs (17). Although bradycardia has been associated with higher survival rates compared to asystole/PEA in pediatric patients (28), we did not observe any difference in survival rate after the 4 h observation period; however, this is likely due to the relatively short period of recovery.

Although the PEA and asystole groups had similarities in the asphyxia duration and blood gas parameters, the ROSC time was about twice of that of the asystole group. Furthermore, there were significantly more piglets that required epinephrine in the PEA group compared to the asystole group, and the number of doses was also significantly greater. Consequently, only 55% of piglets with PEA achieved ROSC. Given the observational nature of this study, our data did not investigate any underlying mechanisms contributing to PEA. Zheng et al. (29) observed a better resuscitation outcome in adult cardiac arrest patients presenting with asystole than those with PEA. They postulated that the pumping activity of the heart was limited in PEA patients during resuscitation due to dissociation of the pumping mechanism, whereas the insufficiency of cardiac output in asystole patients was only due to lack of electrical pacing. Further studies are needed to test this hypothesis in our animal studies. Collectively, our data indicates that heart rhythm influences resuscitation outcome and recovery following CC+SI.

Sobotka et al. (30) described that HR might be a weak indicator for assessing circulatory status in asphyxiated newborns as even with a mean HR of 72 ± 7/min was associated with zero cerebral blood flow in their animal model. In a clinical case series of four neonatal patients, PEA was observed with an electrical HR above 60/min (as displayed on ECG) but either no palpable pulses or a HR assessed by auscultation below 60/min (6). All four patients died after extensive resuscitation, supporting the speculation that PEA is a critical problem in the delivery room. If the HR is only assessed by ECG, this could be misleading and may delay initiation of CCs. A new algorithm approach was described in Luong et al. (6), suggesting using auscultation or palpation as well as ECG and a pulse oximeter for the assessment of a newborn. Doppler ultrasound would be a good alternative to detect PEA, although it is not routinely used (3). Newborns typically present with bradycardia before descending into asystole (28). Therefore, it is necessary to combine ECG monitoring with clinical findings (pulse palpation, auscultation) or pulse oximetry (no pulsatile waveform) for identification of PEA (3). Further studies are needed to assess the prevalence and influence of different heart rhythms in the delivery room.

### Limitations

Our asphyxia model uses piglets that have already undergone the fetal to neonatal transition, and piglets were sedated/anesthetized. Furthermore, our model requires piglets to be intubated with a tightly sealed endotracheal tube to prevent any endotracheal tube leak; this may not occur in the delivery room as mask ventilation is frequently used, in addition to the presence of leak in endotracheally intubated infants. Our experimental protocol differed slightly to the current resuscitation guidelines: we used 100% oxygen after 30 s of CC and administered epinephrine 90 s after CC with a frequency of one dose every 3 min. Furthermore, our baseline values were different between the groups, which are likely due to the slight variability in surgical procedures and anesthetic drugs used. Although we conformed to the 3 R's of animal studies (Replacement, Reduction, and Refinement), we understand that using results of previous studies with different piglet models and different numbers of piglets to perform a secondary analysis also may pose a limitation to data interpretation which were studied using the changes from respective baselines. Further, we acknowledge that as in all animal studies, it may not be possible to translate the results necessarily to human newborns.

## Conclusions

Our study indicates that heart rhythm before resuscitation (bradycardia, asystole, and PEA) influences the time to ROSC, epinephrine administration, and hemodynamic recovery after resuscitation. Evaluation of heart rhythms in the delivery room and their correlation with resuscitation outcome warrants further investigation.

## Data Availability Statement

The raw data supporting the conclusions of this article will be made available by the authors, without undue reservation.

## Ethics Statement

The animal study was reviewed and approved by Animal Care and Use Committee (Health Sciences), University of Alberta.

## Author Contributions

GS conceptualized and designed the study. MW drafted the initial manuscript. MW, T-FL, and GS designed the data collection instruments, collected data, carried out the initial analyses, and reviewed and revised the manuscript. P-YC, MY, VV, and MO'R helped interpreting the results and reviewed and revised the manuscript. All authors approved the final manuscript as submitted and agree to be accountable for all aspects of the work.

## Conflict of Interest

The authors declare that the research was conducted in the absence of any commercial or financial relationships that could be construed as a potential conflict of interest.

## Abbreviations

HR: heart rate
CC: chest compressions
PEA: pulseless electrical activity
ECG: electrocardiogram
C:V: Compression: Ventilation
SI: sustained inflation
ROSC: return of spontaneous circulation
CABF: carotid arterial blood flow.
